# Direct and indirect selection on flowering time, water-use efficiency (WUE, *δ* ^13^C), and WUE plasticity to drought in *Arabidopsis thaliana*

**DOI:** 10.1002/ece3.1270

**Published:** 2014-11-19

**Authors:** Amanda M Kenney, John K McKay, James H Richards, Thomas E Juenger

**Affiliations:** 1 Department of Biological Sciences, St. Edward's UniversityAustin, Texas; 2 Department of Bioagricultural Sciences and Pest Management, Colorado State UniversityFort Collins, Colorado; 3 Land, Air and Water Resources, University of California, DavisDavis, California; 4 Department of Integrative Biology, The University of Texas at AustinAustin, Texas

**Keywords:** *Arabidopsis thaliana*, drought, flowering time, plasticity, selection, water-use efficiency

## Abstract

Flowering time and water-use efficiency (WUE) are two ecological traits that are important for plant drought response. To understand the evolutionary significance of natural genetic variation in flowering time, WUE, and WUE plasticity to drought in *Arabidopsis thaliana*, we addressed the following questions: (1) How are ecophysiological traits genetically correlated within and between different soil moisture environments? (2) Does terminal drought select for early flowering and drought escape? (3) Is WUE plasticity to drought adaptive and/or costly? We measured a suite of ecophysiological and reproductive traits on 234 spring flowering accessions of *A. thaliana* grown in well-watered and season-ending soil drying treatments, and quantified patterns of genetic variation, correlation, and selection within each treatment. WUE and flowering time were consistently positively genetically correlated. WUE was correlated with WUE plasticity, but the direction changed between treatments. Selection generally favored early flowering and low WUE, with drought favoring earlier flowering significantly more than well-watered conditions. Selection for lower WUE was marginally stronger under drought. There were no net fitness costs of WUE plasticity. WUE plasticity (per se) was globally neutral, but locally favored under drought. Strong genetic correlation between WUE and flowering time may facilitate the evolution of drought escape, or constrain independent evolution of these traits. Terminal drought favored drought escape in these spring flowering accessions of *A. thaliana*. WUE plasticity may be favored over completely fixed development in environments with periodic drought.

## Introduction

Water availability significantly limits both natural (Lambers et al. 1998) and crop (Boyer 1982) plant productivity and distribution worldwide. Microhabitat and climatic variation, including water availability, are likely drivers of adaptive differentiation in ecological and physiological traits (Turreson 1922; Stebbins 1952; Lexer and Fay 2005). Furthermore, extensive empirical data indicate that plant populations (Clausen and Heisey 1958; Bennington and McGraw 1995; Dudley 1996a; McKay et al. 2001; Hall and Willis 2006; Lowry et al. 2008; Agren and Schemske 2012) and species (Angert and Schemske 2005; Lexer et al. 2005; Wu and Campbell 2006; Dorman et al. 2009; Campbell et al. 2010) are often locally adapted to environmental conditions (reviewed in Arntz and Delph 2001; Geber and Griffen 2003; Leimu and Fischer 2008; Hereford 2009).

For adaptation to habitats with limited water availability, three broad strategies are described: tolerance, avoidance, and escape (Ludlow 1989). Drought tolerant plants are able to survive low levels of water availability, that is, low soil water potentials. Drought avoiders prevent tissue dehydration by increasing water uptake and/or decreasing water loss, while drought escapers grow during specific seasons and/or complete their life cycle and reproduce before the onset of lethal drought. These adaptive strategies represent multivariate phenotypes and are not mutually exclusive; plants can exhibit traits from more than one strategy (Ludlow 1989). For example, rapid flowering and a high root: shoot ratio may contribute to drought escape and avoidance, respectively. Furthermore, ecophysiological traits are often phenotypically and/or genetically correlated (e.g., Geber and Dawson 1990; Dudley 1996a; McKay et al. 2003; Sherrard and Maherali 2006), which may constrain or facilitate adaptation depending on the direction of selection (Falconer and Mackay 1996; Schluter 1996; Lynch and Walsh 1998). Additionally, selection for local adaptation may often be multivariate, where a particular combination of trait values leads to highest fitness (e.g., Dudley 1996a; Heschel and Riginos 2005; Donovan et al. 2007). Ultimately, elucidating which traits are important for drought response, their functional integration and genetic architecture, and how selection acts on multiple traits across variable environments is central to understanding how different life-histories and drought response strategies evolve (Ackerly et al. 2000; Arntz and Delph 2001; Geber and Griffen 2003; Rausher 2005).

Here, we investigate the evolutionary significance of natural variation in water-use efficiency (WUE) and flowering time. Leaf photosynthetic WUE is the ratio of photosynthetic carbon gain to water loss via transpiration and represents the fundamental trade-off all plants must face—water for carbon (Wong et al. 1979; Farquhar et al. 1989; Geber and Dawson 1990, 1997). WUE can vary by adjustments to photosynthetic rate, stomatal conductance, or concurrent changes in both. In the context of drought response strategies, plants with relatively low WUE that grow rapidly and flower early are described as drought escapers while plants with higher WUE that grow slowly and flower later are described as drought avoiders (Ludlow 1989). It is predicted that high WUE is favorable in consistently water-limited or low competition habitats while lower WUE is favored in wetter or highly competitive environments, or in habitats with terminal drought and/or short growing seasons (Cohen 1970). Likewise, selection on flowering time is hypothesized to favor synchronization with seasonal moisture in dry habitats or early flowering in habitats with short seasons.

Empirical studies often find that selection on WUE and/or gas exchange varies with water availability (Donovan and Ehleringer 1994; Bennington and McGraw 1995; Dudley 1996a; Ludwig et al. 2004; Heschel and Riginos 2005; but see Donovan et al. 2007). Furthermore, selection on WUE may be influenced by other resources, such as soil nitrogen availability (Donovan et al. 2007), or be balanced with selection for increased growth (Bennington and McGraw 1995). Consistent with drought escape, water-limited conditions often favor earlier flowering (e.g., Bennington and McGraw 1995; Heschel and Riginos 2005; Franks et al. 2007; Franks 2011; Ivey and Carr 2012; but see Sherrard and Maherali 2006). Plant species and populations often show genetically based phenotypic differentiation consistent with patterns of differential selection and/or predictions of climatic adaptation (e.g., Dudley 1996a,b; McKay et al. 2001; Heschel et al. 2002; Franks et al. 2007; Lowry et al. 2008; Wu et al. 2010; Franks 2011). Additionally, WUE and flowering time are often positively correlated (e.g., McKay et al. 2003; Sherrard and Maherali 2006); however, this is not always the case (Ivey and Carr 2012) and may depend on the scale of comparison (Wu et al. 2010) or developmental stage (Sherrard and Maherali 2006).

In addition to being genetically variable, ecophysiological traits (including water-use and reproductive traits) are highly plastic in response to many environmental variables, for example, water availability (e.g., Heschel et al. 2002; Hausmann et al. 2005; Sherrard and Maherali 2006; Wu et al. 2010), temperature (Stinchcombe et al. 2004a), and conspecific plant density (Weinig et al. 2006). Phenotypic plasticity is often assumed to be adaptive; however, the potential costs of plasticity may outweigh the benefits (Tienderen 1991; DeWitt et al. 1998; van Kleunen and Fischer 2005, 2007). Additionally, the costs and benefits of plasticity may be limited to specific environments or have global effects on fitness across all environments (DeWitt et al. 1998; van Kleunen and Fischer 2005). It is not always clear whether selection acts directly on plasticity per se or if selection acts indirectly on plasticity through direct selection on phenotypic trait values within different environments (Via 1993; Via et al. 1995). Both processes probably occur, but the relative importance of each is likely determined by multiple factors, for example, the scale of environmental heterogeneity, potential costs of plasticity, level of migration between habitats, and clonality/family structure (Via et al. 1995; Sultan and Spencer 2002). For example, selection may favor greater plasticity per se within populations that grow in highly heterogeneous, fine-grained environments, but favor more fixed trait expression within populations in less heterogeneous environments (e.g., Baythavong 2011). Moreover, experiments measuring variation in phenotypic plasticity and its relationship to fitness in and across environments (e.g., Schmitt et al. 1999, 2003; Stinchcombe et al. 2004a; Caruso et al. 2006; Weinig et al. 2006; Maherali et al. 2010; Baythavong 2011) can directly test whether the net effect of plasticity is adaptive, neutral, or costly (Tienderen 1991; DeWitt et al. 1998; van Kleunen and Fischer 2005, 2007; Auld et al. 2010).

*Arabidopsis thaliana* is a classic model system for studying molecular genetics, genomics, quantitative genetics, stress response, physiological variation (reviewed in Alonso-Blanco and Koornneef 2000; Borevitz and Nordborg 2003; Koornneef et al. 2004; Mitchell-Olds and Schmitt 2006; Koornneef and Meinke 2010; Verslues and Juenger 2011; Assmann 2013; Juenger 2013), and more recently, local adaptation (Fournier-Level et al. 2011; Hancock et al. 2011; Agren and Schemske 2012). Populations of *A. thaliana* are locally adapted to their respective environments (Agren and Schemske 2012), and climate is an important force shaping adaptive genomic variation (Fournier-Level et al. 2011; Hancock et al. 2011; Banta et al. 2012; Lasky et al. 2012) and patterns of gene polymorphism (Lee and Mitchell-Olds 2012). *A. thaliana* populations are extremely genetically variable for flowering time (McKay et al. 2003; Caicedo et al. 2004; Stinchcombe et al. 2004b; Aranzana et al. 2005; Juenger et al. 2005b; Atwell et al. 2010; Brachi et al. 2010; Grillo et al. 2013) which is a key component of fitness (Korves et al. 2007) and adaptation to climate (Fournier-Level et al. 2011; Hancock et al. 2011). WUE is also genetically variable among populations (Nienhuis et al. 1994; McKay et al. 2003), and it appears divergence in WUE may be adaptive for drought response among different habitats (McKay et al. 2003; Lovell et al. 2013). Furthermore, these two traits are positively genetically correlated among natural accessions across the geographic range of *A. thaliana* (McKay et al. 2003; Lovell et al. 2013), and multiple QTL (Hausmann et al. 2005; Juenger et al. 2005a) and genes (McKay et al. 2003; Kinoshita et al. 2011; Lovell et al. 2013) pleiotropically affect both flowering time and WUE (and/or physiological processes that affect WUE). Despite this previous work, we do not know how variation in WUE and flowering time is related to plant growth or whether the genetic correlations among ecophysiological traits are affected by water availability in *A. thaliana*. Additionally, how selection acts on WUE and flowering time across different moisture environments, as well as the evolutionary implications of WUE plasticity to drought, is unclear.

Here, we combine experimental manipulation, quantitative genetics, and genetic selection analyses to address the evolutionary significance of natural genetic variation in flowering time, WUE, and WUE plasticity to drought in *Arabidopsis thaliana*. Specifically, we focus on the following questions: (1) How are ecophysiological traits genetically correlated within and between different moisture environments? (2) Does terminal drought select for early flowering and escape? and (3) Is WUE plasticity to drought adaptive and/or costly?

## Materials and Methods

### Plant material

We studied 234 natural accessions (Table S1) of the annual herb *Arabidopsis thaliana* (L.) Heyhn. (Brassicaceae) to explore plant growth, physiology, and reproduction in response to terminal drought. We obtained the majority of accessions directly from the Arabidopsis Biological Resources Center at The Ohio State University. In addition, a small number of lines were provided through the generosity of individual researchers. Our analysis exclusively includes “spring” accessions lacking a strong vernalization requirement to flower under our greenhouse growing conditions. We note that the flowering time and WUE data from a majority of these lines in the well-watered treatment (see below for treatment and phenotype details) also appear in Lovell et al. (2013).

### Growth conditions and experimental design

Replicate plants from each accession were grown under standard greenhouse conditions using Promix BT potting soil™ and 164-mL Cone-tainers™ (Stuewe and Sons, Tangent, Oregon, USA). Several seeds were initially planted into each Cone-tainer™ and subsequently thinned at the first true leaf stage to a single replicate individual per pot. Individual Cone-tainers™ were organized in 2 × 1-ft. trays at half the possible density (49 plants per tray, skipping every other position). Seeds were cold stratified at 4°C for 5 days in a walk-in environmental chamber, then transferred to a greenhouse with long-day photoperiod conditions (16 h light/8 h dark). Light levels were maintained above a minimum of 1000  *μ* mol m^−2^ s^−1^ with supplemental light provided by 600 watt high-intensity discharge lamps as needed. Greenhouse temperature was maintained at *ca*. 18–21°C. Plants were tended daily and hand-watered with a spray wand.

To study plant responses to water availability, we altered the watering regime to create two treatments—either a long growing season where plants were well watered for 6 weeks, or terminal drought where plants were water-limited after 4 weeks. Both groups of plants were treated identically until week 4, when watering ceased in the terminal drought treatment. In general, the complete dry down of pots was slow and corresponded to approximately 8 days. At the time of treatment initiation, ∼70% of the accessions had begun flowering. Thus, our terminal drought treatment mimics a drought that occurs in the middle of, and effectively truncates, the growing season. The earliest plants flowered after approximately 2 weeks in the greenhouse, leading to an overall period of ∼4 weeks of flowering time initiation among the accession panel. At treatment initiation, all plants were green and flowering individuals were still producing new flowers and fruits. At the end of the experiment, most plants had completed flowering and many were senescing.

We note that the timing of the treatment initiation relative to the span of flowering initiation limits some aspects of our analyses and conclusions (see Results and Discussion). Our experiment specifically mimics a season-ended drought, where the growing season is truncated and there is some variation in flowering time within a population. Although imperfect, applying the treatment after/before some lines have initiated flowering allows all the plants to experience the drought while they are still growing, rather than some plants completing their life cycle before the drought (if drought occurred after all plants flowered) or many plants dying and never getting a chance to flower (if drought occurred before all plants flowered).

We assessed the effect of drought using a fully factorial randomized block design with accession and treatment as experimental factors. The two levels of the irrigation treatment (long season and terminal drought) were applied at the level of individual pots. In total, ∼2340 plants were evaluated for responses to the irrigation treatment (234 lines × 2 treatments × 5 replicates = 2340). The experiment was planted in late November and harvested in early January.

### Phenotypic measurements

For each experimental plant, we recorded the rank ordered date of first flowering, aboveground dry biomass at harvest, the final number of fruits (siliques) at harvest, and an estimate of average fruit length per plant (mean of 3 haphazardly chosen fruits). Fruit length and number of ovules (or seeds) per fruit have been shown to be positively correlated in *A. thaliana* (Alonso-Blanco et al. 1999); therefore, we considered the product of final fruit number and average fruit length as an estimate of lifetime reproductive fitness (total fruit length; also used in Hausmann et al. 2005). Flowering time was recorded through daily inspection of the plants and was scored upon the observation of the first open flower bud. We transformed the calendar date of first flowering to an ordered quantitative trait by assigning the first flowering day in the experiment a value of one.

#### Water-use efficiency (WUE) estimates

We estimated integrated WUE as the carbon isotopic composition (*δ* ^13^C) of aboveground biomass of all accessions (Farquhar et al. 1989; Lambers et al. 1998; Dawson et al. 2002; McKay et al. 2003; Juenger et al. 2005a). We used *δ* ^13^C rather than Δ^13^C because of the variability of the source CO_2_ *δ* ^13^C in the greenhouse. We used a pooling scheme to derive an independent point estimate of *δ* ^13^C for each accession in each treatment. At the end of the experiment, the aboveground material from all available replicate plants from each accession was pooled and course ground in centrifuge tubes, after which subsamples were fine ground in microcentrifuge tubes with ball bearings. Two mg of finely ground tissue was loaded into a tin capsule and analyzed at the UC Davis Stable Isotope Facility (http://stableisotopefacility.ucdavis.edu). Data are presented as carbon isotope ratios relative to the V-PDB standard (*R*_PDB_), where *δ* ^13^C (‰) = (*R*_sample_/R_PDB_−1)*1000. These values are expressed per mil (‰).

### Data analysis

#### Quantitative genetic analyses

To determine the significance of each experimental factor's contribution to the variance in each measured trait, we performed linear mixed model analyses using Proc Mixed in SAS (SAS/STAT® software version 9.2, Littell et al. 2006). Unless otherwise noted, all subsequent analyses were performed using Proc Mixed. Accession, accession*treatment, block, and tray nested within block were treated as random effects. Treatment was analyzed as a fixed effect and tested for significance using an *F* -ratio test. Variance components were estimated for random effects using restricted maximum likelihood (REML; Lynch and Walsh 1998). Individual components were tested for significance using likelihood ratios tests comparing a full model to one with that single component removed. A significant treatment effect indicates there is significant plasticity to the drought treatment. A significant among-accession variance component indicates there is significant genetic variance (*V*_*g*_). A significant accession*treatment variance component indicates there is significant genetic variation for plastic response to terminal drought. Variance components were not estimated for *δ* ^13^C because the replicates within treatment were pooled for a point estimate of WUE for each accession. We tested for a significant fixed effect of the drought treatment on *δ* ^13^C using an *F* -ratio test.

To estimate quantitative genetic parameters separately for plants growing within long season and terminal drought conditions, we estimated variance components within each treatment. Accession, block, and tray nested within block were treated as random effects. Here, the among-accession variance component (*V*_*g*_) is an estimate of quantitative genetic variation within each environment. We calculated broad-sense heritability (H^2^) by dividing the genetic variance by the total phenotypic variance, that is, the sum of all variance components (H2 = Vg/Vp; Lynch and Walsh 1998). Note that broad-sense heritability in this context includes additive effects and epistasis, but that dominance variation is likely minimal due to high homozygosity in *A. thaliana*.

To produce estimates of breeding values for each accession within each treatment, we generated least squares means (LSMeans) using Proc Mixed for each accession-by-treatment combination. Satterthwaite degrees of freedom were specified in the LSMeans statement. We considered the pooled estimate of *δ* ^13^C for each accession-by-treatment combination a breeding value for WUE.

To estimate genetic correlations between traits, we calculated the Pearson's product–moment correlation coefficient between the breeding values for all pairwise combinations of measured traits using Proc Corr (SAS/STAT). As a quantification of variation in phenotypic plasticity, we calculated the genetic correlation between treatments for each trait. Values significantly less than 1 demonstrate genetic variation in plasticity (Lynch and Walsh 1998). 95% confidence intervals around all correlation coefficients were estimated from 5000 replicate bootstrap samples using the Boot package in R (sampled with replacement, intervals are from “basic bootstrap intervals”; Canty 2002; R Core Team 2013).

We calculated WUE (*δ* ^13^C) plasticity as the difference in *δ* ^13^C between the long season and drought treatments. Long season values were subtracted from drought values (plasticity = drought − long) such that all WUE plasticity values represent the response to the drought treatment relative to the long season treatment. These signed difference values were used for estimating genetic correlations and selection on WUE plasticity so that analyses would correctly distinguish between increases and decreases in trait values across treatments (van Kleunen and Fischer 2005, 2007).

#### Genetic selection analyses

To gain a comprehensive understanding of how selection may act within and across different environments, we performed several univariate and multivariate genetic selection analyses. First, to estimate total selection (i.e., direct + indirect selection), we calculated directional selection differentials for each trait within each treatment as the regression coefficient of relative fitness regressed on an individual trait (Lande and Arnold 1983). Analyses were performed using standardized LSMeans of phenotypic traits as genetic breeding values (accession means for *δ* ^13^C). Phenotypic traits, including *δ* ^13^C plasticity, were standardized to a mean of zero and unit variance within each treatment [(individual value − treatment mean)/treatment standard deviation]. Relative fitness was calculated by relativizing the LSMeans of absolute fitness to the mean within each treatment (individual value/treatment mean). Regression coefficients, standard errors, and *P* -values for nonzero significance tests were generated using Proc Mixed. 95% confidence intervals around regression coefficients were estimated as above for genetic correlations.

Second, we calculated multivariate direct selection gradients within each treatment as the partial regression coefficients from a multiple regression of relative fitness on all measured traits (Lande and Arnold 1983). Fitness and phenotypic trait values were relativized and standardized as above. Linear selection was determined from models with main effects only. Nonlinear and correlational selection gradients were estimated by adding all trait*trait terms to the multiple regression model. Nonlinear regression coefficients, standard errors, and confidence intervals were doubled to produce correct nonlinear selection gradients (Stinchcombe et al. 2008).

Finally, for comparability to other studies that estimate the benefits and costs of plasticity, we performed two kinds of modified analyses to estimate selection on WUE (*δ* ^13^C) plasticity to drought. First, we estimated local selection on plasticity by modeling relative fitness as a function of WUE (*δ* ^13^C) and plasticity within each treatment (DeWitt et al. 1998; van Kleunen and Fischer 2005). WUE (*δ* ^13^C) and fitness were standardized and relativized within treatment, respectively. Second, we estimated global selection on WUE (*δ* ^13^C) plasticity by modeling relative fitness across treatments as a function of average WUE value and WUE plasticity (van Kleunen and Fischer 2005). For this analysis, *δ* ^13^C values in the long season and drought treatments were averaged for each accession then standardized to a mean of zero and standard deviation of 1. Fitness was averaged across treatments then relativized to the global mean. Linear selection was determined from models with main effects only and correlational selection was determined from models with the trait*plasticity interaction term added.

To determine whether selection on the same trait was significantly different between treatments, we performed *t* -tests (Sokal and Rohlf 1995). This specifically tests whether the proportional change in relative fitness as a function of change in standardized trait value is different between treatments. For multivariate selection, this specifically tests whether the proportional change in fitness after accounting for the effects of other traits is different between treatments.

## Results

### Plasticity to drought and genetic variation

**Figure 1:**
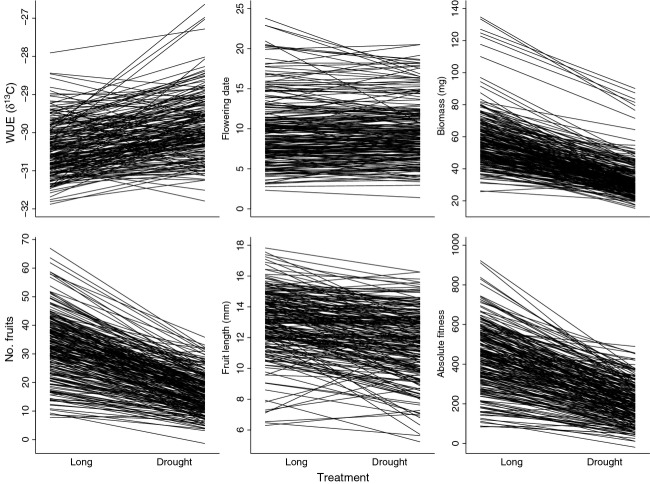
Plastic response of *Arabidopsis thaliana* to terminal drought. Values are least squared means for all individually measured traits within the long season and drought treatments. WUE (*δ* ^13^C) values are the pooled accession values within each treatment. Flowering date = rank ordered flowering date. Biomass = final dry biomass. No. fruits = number of fruits at harvest. Fruit length = average length of three haphazardly measured fruits per plant. Absolute fitness = fruit number multiplied by fruit length. Two fruit length data points (one extremely high, one low) are not shown to improve clarity of slopes of the remaining lines.

We detected a significant response to terminal drought for growth, physiology, and fitness (Table1, Fig.1). In general, most plants in the drought treatment had higher WUE (higher *δ* ^13^C, *F*  = 47.44, *P*  < 0.0001), lower biomass, made fewer and smaller fruits, and had lower absolute fitness than plants in the long season treatment (Table2, Fig.1). These plastic changes are similar to other studies on drought response in *A. thaliana* (e.g., Hausmann et al. 2005) and other species (e.g., Heschel et al. 2002; Sherrard and Maherali 2006; Wu et al. 2010). *δ* ^13^C plasticity ranged from −1.75 to 3.35 (mean = 0.55 higher *δ* ^13^C under drought), with ∼79% of the accessions having positive plasticity values. The reduction of fitness in the terminal drought treatment was due more to reduced fruit number than reduced fruit length (Table2, Fig.1). We note that we included flowering time in the standard quantitative analyses in Table1 for consistency, but that due to the timing of the treatment, we did not expect a strong effect of drought on flowering initiation. For this reason, all other analyses and discussions of plastic response focus on WUE and the other measured traits and do not include flowering time.

**Table 1:** Genetic and environmental effects on phenology, growth, and fitness

Trait	Flowering date	Biomass	No. fruits	Fruit length	Absolute fitness
Random effects					
Accession	14.67***	152.58***	56.16***	3.73***	11,405.00***
Accession × Treatment	0.28*	29.99***	16.32***	0.53***	3842.54***
Block	0.20***	4.35*	3.36***	0.19***	1012.99***
Tray (Block)	0.08*	15.33***	2.28***	0.11***	478.55***
Residual	4.31	161.14	56.97	2.70	12,515.00
Fixed effects					
Treatment					
*F-ratio*	2.00	1084.03***	1115.57***	188.76***	992.30***
df	1208	1230	1227	1213	1229

Variance component estimates are provided for random effects. *F* -ratios and degrees of freedom are provided for fixed effects. Absolute fitness = number of fruits × fruit length.

*** *P*  < 0.0001;

* *P*  ≤ 0.05.

**Table 2:** Mean and variability for each trait within the long season and drought treatments

	Mean (SE)				Range		*V*_*g*_		*V*_*p*_		H^2^	
	Long season		Drought		Long season	Drought	Long season	Drought	Long season	Drought	Long season	Drought
*δ* ^13^C	−30.37	(0.05)	−29.86	(0.06)	−31.88, −27.91	−31.80, −26.64						
Flowering date	9.57	(0.14)	9.10	(0.12)	1, 28	1, 24	16.29	12.65	21.17	16.87	0.77	0.75
Biomass (mg)	58.23	(0.67)	33.63	(0.45)	3.0, 184.1	3.6, 119.6	246.68	121.12	500.24	226.46	0.49	0.53
No. fruits	33.25	(0.41)	16.88	(0.27)	0, 102	0, 53	103.18	40.31	188.38	78.25	0.55	0.52
Fruit length (mm)	13.14	(0.08)	11.91	(0.08)	0, 27	0, 23	4.09	4.63	6.83	7.88	0.60	0.59
Absolute fitness	443.06	(6.07)	210.27	(3.94)	0, 1278	0, 690	21,371.0	8765.0	40,700.1	17,030.2	0.53	0.51

Mean (±SE), range (min, max), genetic variance (*V*_*g*_), total phenotypic variance (*V*_*p*_), and broad-sense heritability (H^2^ =  *V*_*g*_/ *V*_*p*_) are given for each individually measured trait. For individually measured traits, means and ranges are from individual plant values, that is, not line means. For *δ* ^13^C, the mean (±SE) and range are from the pooled accession values within treatment.

We detected a significant among-accession variance component for all individually measured traits (Table1, Fig.1), demonstrating quantitative genetic variation for flowering time, growth, and fitness. Accession-by-treatment interactions explained a significant proportion of the phenotypic variance for all traits (Table1), demonstrating drought differentially affected trait expression for different accessions, that is, there is genetic variation for plasticity to drought. Broad-sense heritability (H^2^) was moderate (range: 0.49–0.77) for all traits across both treatments (Table2). Total phenotypic variance (*V*_*p*_) was generally greater in the wet treatment for most traits, with H^2^ being relatively similar across treatments (Table2). Accession pooling precluded variance component analyses and heritability estimates of *δ* ^13^C; however, the range of *δ* ^13^C values was biologically and physiologically significant based on previous work (McKay et al. 2003; Juenger et al. 2005a), indicating there is likely genetic variation for WUE.

### Genotypic correlations

**Figure 2:**
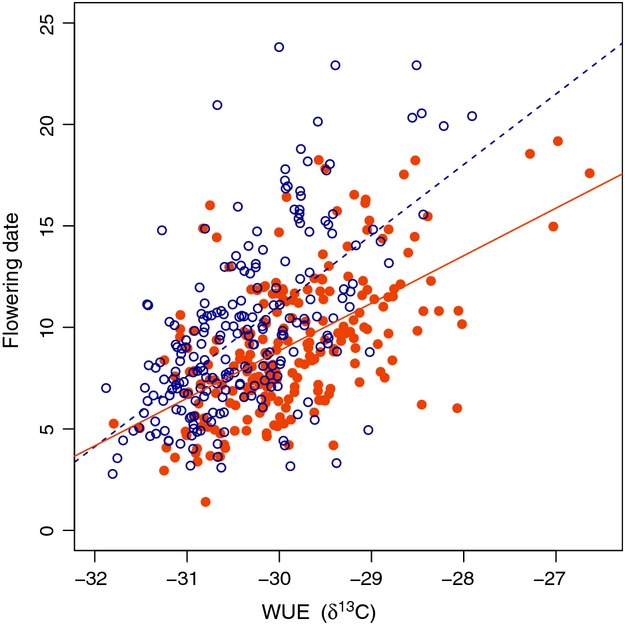
Positive genetic correlation between WUE (*δ* ^13^C) and flowering time among spring annual accessions of *Arabidopsis thaliana*. Genetic correlations within the long season (blue, open circles, and dashed line) and drought (orange, closed circles, and solid line) treatments are *r*  = 0.59 and *r*  = 0.57, respectively (*P*  < 0.0001 for both). Flowering time values are the least squared means within each treatment. WUE (*δ* ^13^C) values are the pooled accession values within each treatment. Best-fit regression equations are *Y*  = 3.40 *X*  + 115.4 in the long season (*R* ^2^ = 0.32, *P*  < 0.0001) and *Y*  = 2.34 *X*  + 79.1 in the drought treatment (*R* ^2^ = 0.35, *P*  < 0.0001).

*δ* ^13^C was positively correlated with flowering time within both treatments (lower WUE correlated with earlier flowering; Fig.2) and negatively correlated with fitness (lower WUE correlated with higher fitness; Table3a). Within both treatments, the greatest correlation was a negative relationship between flowering time and fitness (earlier flowering correlated with higher fitness), a result that is mirrored in our selection analyses below. Overall, patterns of among-trait genotypic correlations were similar within both treatments, demonstrating that drought did not drastically alter the relationships between traits (Table3a). One difference between treatments included a significant negative correlation between flowering time and biomass in the drought treatment (earlier flowering correlated with greater biomass), but no correlation in the long season treatment.

**Table 3:** Genotypic correlations within (a) and between (b) treatments

	*δ* ^13^C		*δ* ^13^C Plasticity		Flowering date		Biomass		Absolute fitness	
(a) Between traits, within treatment										
*δ* ^13^C			−0.37*** (−0.51, −0.24)	**L**	0.59*** (0.50, 0.71)	**L**	0.07 (−0.05, 0.21)	L	−0.43*** (−0.57, −0.31)	**L**
*δ* ^13^C plasticity	0.61*** (0.51, 0.73)	**D**			0.06 (−0.10, 0.21)	L	0.03 (−0.10, 0.15)	L	0.03 (−0.13, 0.19)	L
Flowering date	0.57*** (0.47, 0.68)	**D**	0.14* (−0.04, 0.32)	**D**			−0.06 (−0.18, 0.05)	L	−0.64*** (−0.72, −0.56)	**L**
Biomass	0.01 (−0.13, 0.16)	D	0.05 (−0.09, 0.18)	D	−0.32*** (−0.41, −0.24)	**D**			0.59*** (0.50, 0.69)	**L**
Absolute fitness	−0.47*** (−0.59, −0.37)	**D**	−0.07 (−0.23, 0.08)	D	−0.79*** (−0.83, −0.74)	**D**	0.57*** (0.48, 0.67)	**D**		
	*r*	95% CI								
(b) Same trait, between treatments										
*δ* ^13^C versus *δ* ^13^C	0.51***	(0.41, 0.62)								
Biomass versus Biomass	0.75***	(0.67, 0.87)								
Absolute fitness versus Absolute fitness	0.70***	(0.64, 0.76)								

Values above and below the diagonal in (a) represent correlations within the long season (L) and drought (D) treatments, respectively. *N*  = 206–234. Values are Pearson's product–moment correlation coefficients. Values in parentheses are 95% confidence intervals.

Significant values are in bold.

*** *P*  < 0.0001;

* *P*  ≤ 0.05.

Within-trait between-treatment correlations were all positive and significantly less than 1, and ranged from *r*  = 0.51 to 0.75 (Table3b), demonstrating that genetic variation in plasticity varies among traits. *δ* ^13^C had the greatest variation in plasticity to drought (lowest correlation between treatments; *r*  = 0.51).

#### Genotypic correlations between WUE (*δ* ^13^C) plasticity and phenotypic traits

**Figure 3:**
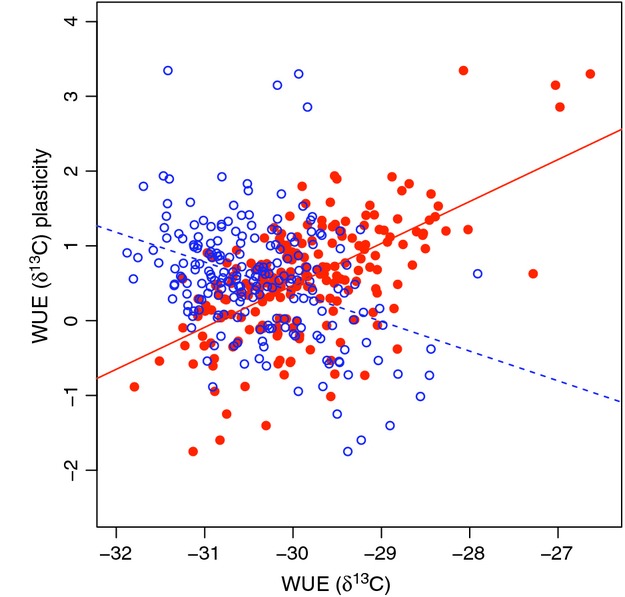
Genetic correlation between WUE (*δ* ^13^C) and WUE plasticity to drought within the long season (blue, open circles, and dashed line) and drought (orange, closed symbols, and solid line) treatments. Flowering time values are the least squared means within each treatment. WUE (*δ* ^13^C) values are the pooled accession values within treatment. Genetic correlations are *r*  = −0.37 and r = 0.61 (*P*  < 0.0001 for both) for the long season and drought treatments, respectively. Best-fit regression equations are *Y*  = −0.40 *X*  − 11.55 (*R* ^2^ = 0.13, *P*  < 0.0001) in the long season treatment, and *Y*  = 0.56 *X*  + 17.30 (*R* ^2^ = 0.37, *P*  < 0.0001) in the drought treatment.

Accessions with the greatest WUE plasticity (either increase or decrease in WUE) had the most extreme values of WUE in both the long season and drought treatments (Fig.3). For example, WUE plasticity was positively correlated with higher WUE in the drought treatment; accessions with the highest WUE values under drought also had the greatest increase in WUE from long season to drought, while plants with the lowest WUE under drought showed a decrease in WUE from long season to drought (Fig.3). This relationship was reversed when considering WUE in the well-watered long season treatment. WUE plasticity was negatively correlated with WUE in the long season treatment; accessions with lower WUE in the long season treatment also had the greatest increase in WUE from the long season to drought (and the highest WUE under drought). Likewise, the accessions with the highest WUE in the long season treatment had the lowest WUE in the drought treatment (Fig.3). It seems there were two classes of plastic response to drought: accessions with increased WUE under drought (155 lines/75.2% with ≥0.1 *δ* ^13^C increase) and accessions with decreased WUE under drought (36 lines/17.5% with ≥0.1 *δ* ^13^C decrease; Fig.1). It may be that plasticity for increased WUE reflects physiological adjustments to conserve water (e.g., decreased stomatal conductance) while decreased WUE under drought reflects physiological adjustments to hasten development or less efficient photosynthesis due to limited resources. WUE plasticity was also positively correlated with mean between-treatment WUE (*r*  = 0.18, *P*  = 0.009); accessions with higher mean WUE overall had greater plastic increase in WUE from the long season to drought. WUE plasticity was marginally, positively correlated with flowering date in the drought treatment (accessions with greater plastic increase in WUE flowered later) but not in the long season treatment (Table3).

### Selection in long season versus drought conditions

#### Total selection—selection differentials

**Figure 4:**
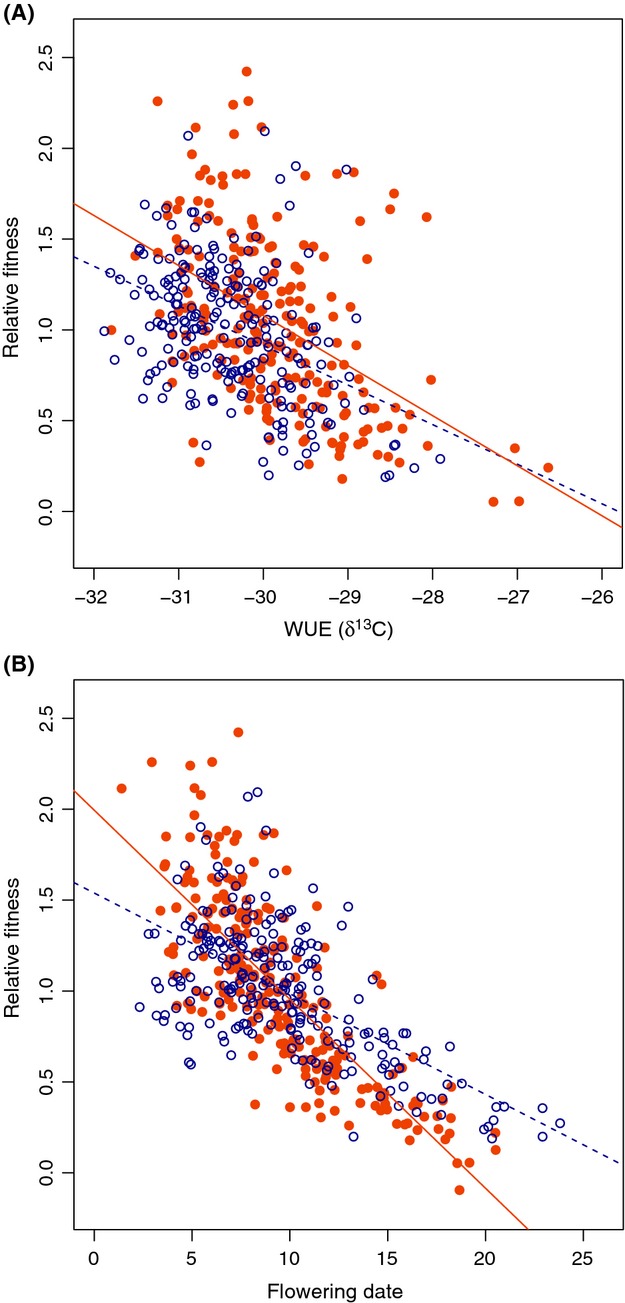
Relative fitness as a function of WUE (*δ* ^13^C; A) and flowering time (B) within the long season (blue, open circles, and dashed line) and drought (orange, solid circles, and solid line) treatments. Flowering time values are the least squared means within each treatment. WUE (*δ* ^13^C) values are the pooled accession values within treatment. Relative fitness = least squared means of absolute fitness relativized to the mean within each treatment. Best-fit regression equations for WUE are *Y*  = −0.28 *X*  − 7.19 (*R* ^2^ = 0.22, *P*  < 0.0001) in the drought treatment, and *Y*  = −0.22X − 5.63 (*R* ^2^ = 0.18, *P*  < 0.0001) in the long season treatment. Best-fit regression equations for flowering time are *Y*  = −0.10 *X*  + 1.99 (*R* ^2^ = 0.62, *P*  < 0.0001) in the drought treatment and *Y*  = −0.05 *X*  + 1.54 (*R* ^2^ = 0.40, *P*  < 0.0001) in the long season treatment.

We detected total selection (i.e., direct + indirect) on all traits, except WUE (*δ* ^13^C) plasticity, as significant selection differentials in both the long season and drought treatments (Table4a). The direction of total selection on mean trait values was the same across environments with selection favoring lower WUE (lower *δ* ^13^C; Fig.4A), earlier flowering (Fig.4B), and greater biomass. Within both treatments, flowering time was under the strongest selection (Table4a). The strength of total selection on both flowering date and biomass was significantly greater under drought than in the long season treatment (Table4a). Total selection for lower WUE was marginally stronger in the drought treatment (Table4a).

**Table 4:** Total (a) and direct (b) selection within the long season and drought treatments

(a) Total selection—Selection differentials															
Trait	Long season						Drought						Test for *s* difference		
	*s*	SE	95% CI	df	*F*	*P*	*s*	SE	95% CI	df	*F*	*P*	*t*	df	*P*
*δ* ^13^C	−**0.159**	0.023	(−0.199, −0.106)	1, 220	49.61	<0.0001	−**0.231**	0.029	(−0.284, −0.169)	1, 216	62.90	<0.0001	1.95	424	0.051
*δ* ^13^C plasticity	0.012	0.025	(−0.046, 0.068)	1, 204	0.22	0.639	−0.035	0.034	(−0.109, 0.037)	1, 204	1.10	0.296	–	**–**	–
Flowering date	−**0.232**	0.018	(−0.263, −0.200)	1, 232	158.02	<0.0001	−**0.394**	0.020	(−0.432, −0.358)	1, 232	372.78	<0.0001	5.88	464	**<0.0001**
Biomass	**0.216**	0.019	(0.161, 0.254)	1, 232	124.68	<0.0001	**0.286**	0.027	(0.204, 0.363)	1, 232	111.81	<0.0001	2.12	464	**0.034**
(b) Direct selection—Selection gradients															
Trait	Long season						Drought						Test for *β* difference		
	*β*	SE	95% CI	df	*F*	*P*	*β*	SE	95% CI	df	*F*	*P*	*t*	df	*P*
*δ* ^13^C	−**0.072**	0.019	(−0.108, −0.037)	1, 201	14.98	<0.001	−**0.124**	0.028	(−0.176, −0.074)	1, 201	19.27	<0.0001	1.53	402	0.126
*δ* ^13^C plasticity	−0.011	0.015	(−0.040, 0.021)	1, 201	0.53	0.468	**0.061**	0.023	(0.024, 0.105)	1, 201	7.10	0.008	2.63	402	**0.009**
Flowering date	−**0.172**	0.018	(−0.205, −0.135)	1, 201	89.33	<0.0001	−**0.258**	0.026	(−0.310, −0.207)	1, 201	100.82	<0.0001	2.73	402	**0.007**
Biomass	**0.231**	0.013	(0.198, 0.263)	1, 201	293.48	<0.0001	**0.218**	0.019	(0.168, 0.266)	1, 201	138.12	<0.0001	0.58	402	0.576

Standardized linear selection differentials (s; *N*  = 206–234) and gradients (*β*; *N*  = 206 for both treatments) in the long season and drought treatments. Significant selection differentials and gradients, and significant *P* -values for the difference between treatments, are in bold.

#### Direct selection—multivariate selection gradients

Selection gradients from multivariate analyses of fitness demonstrate significant direct linear selection on all mean trait values in both treatments (Table4b). Direct selection favored lower WUE (*δ* ^13^C), earlier flowering, and greater biomass in both treatments. Similarly to total selection, direct selection for earlier flowering was significantly higher in the drought treatment (Table4b). There was a trend for stronger selection for lower WUE in the drought treatment, but this was not significant (Table4b). Selection favored greater WUE (*δ* ^13^C) plasticity in the drought treatment, while there was no direct selection on WUE plasticity in the long season treatment (Table4b). In contrast to total selection, direct selection on biomass was not significantly different between treatments (Table4b).

Neither nonlinear nor correlational selection were major modes of selection (Table S2). One pattern was a positive, nonlinear selection gradient for flowering date in the drought treatment (*γ*_ii_ = 0.148, *P*  = 0.009), resulting in a nonlinear increasing function of fitness with earlier flowering time. There was weak evidence for nonlinear direct selection on biomass (*γ*_ii_ = −0.085) and correlational selection on WUE plasticity and biomass (*γ*_ij_ = 0.053; Table S2) under drought.

### Global versus local selection on WUE (*δ* ^13^C) plasticity

We detected global linear selection for lower WUE (lower *δ* ^13^C), but no global selection for or against WUE plasticity and no global selection on the interaction between WUE and WUE plasticity (Table5b). In contrast, we detected significant local selection for greater WUE plasticity (larger plastic increase in WUE under drought) within the drought treatment, but not the long season treatment (Table5a). No local selection on the interaction of WUE and WUE plasticity was detected in either treatment. This suggests that plasticity was globally neutral, but that selection favored greater plastic development per se within the drought treatment. Additionally, separating out selection on WUE and WUE plasticity yielded significantly different selection gradients on WUE between the long season and drought treatments (Table5a), suggesting stronger selection for lower WUE under drought after accounting for correlations with plasticity.

**Table 5:** Local (a) and global (b) selection on WUE (*δ* ^13^C) and WUE plasticity

(a) Local selection															
Trait	Long season						Drought						Test for *β* difference		
	*β*	SE	95% CI	df	*F*	*P*	*β*	SE	95% CI	df	*F*	*P*	*t*	df	*P*
*δ* ^13^C	−**0.147**	0.025	(−0.201, −0.088)	1, 203	33.39	<0.0001	−**0.322**	0.037	(−0.386, −0.246)	1, 203	77.61	<0.0001	3.94	406	**<0.0001**
*δ* ^13^C plasticity	−0.041	0.025	(−0.092, 0.011)	1, 203	2.74	0.100	**0.160**	0.036	(0.086, 0.228)	1, 203	19.23	<0.0001	4.56	406	**<0.0001**
*δ* ^13^C^*^ *δ* ^13^C plasticity (*γ*_ij_)	−0.005	0.022	(−0.048, 0.042)	1, 202	0.06	0.807	−0.013	0.019	(−0.045, 0.030)	1, 202	0.46	0.498	–	–	–
(b) Global selection															
Trait	*β*	SE	95% CI	df	*F*	*P*									
*δ* ^13^C	−**0.174**	0.023	(−0.218, −0.121)	1, 203	57.51	<0.0001									
*δ* ^13^C plasticity	0.029	0.023	(−0.017, 0.072)	1, 203	1.55	0.214									
*δ* ^13^C^*^ *δ* ^13^C plasticity (*γ*_ij_)	−0.017	0.019	(−0.050, 0.024)	1, 202	0.77	0.382									

Standardized linear (*β*) and correlational (*γ*_ij_) selection gradients locally within and globally across treatments (*N*  = 206 for all). Significant selection gradients and significant *P* -values for the difference between treatments are in bold.

## Discussion

### Genetic variation in ecophysiological traits and potential for evolutionary response to selection

We detected significant genetic variation for all individually measured traits in both treatments, including fitness, in our panel of *A. thaliana* accessions as significant genetic variance components and moderate broad-sense heritabilities. The range of WUE (*δ* ^13^C = −31.80 to −26.64 under drought, more than five *δ* ^13^C units) was large and biologically significant. In *A. thaliana*, a 0.5 increase in *δ* ^13^C can correspond to a 25% increase in transpiration efficiency (biomass gained/water transpired; Juenger et al. 2005a). These results demonstrate there is substantial genetic variation for important ecophysiological traits and fitness among *Arabidopsis* accessions and potential for future evolutionary response to selection (Falconer and Mackay 1996; Lynch and Walsh 1998).

### Genetic correlation as adaptive facilitation or constraint?

We found highly significant genetic correlations between multiple ecophysiological traits, including fitness, within both treatments. Of particular note is the consistent, positive genetic correlation between WUE and flowering time, which may facilitate adaptation as a “line of least resistance” to the evolution of fast escapers and/or late avoiders (Schluter 1996). However, this may also act as an evolutionary constraint to the evolution of different combinations of traits, for example, high WUE and early flowering (Falconer and Mackay 1996; Schluter 1996; Lynch and Walsh 1998). The extent to which this genetic correlation is due to pleiotropy, linkage disequilibrium, and/or genetic linkage will likely determine the potential for future independent evolution of WUE and flowering time (Falconer and Mackay 1996; Lynch and Walsh 1998). Furthermore, correlations with additional performance traits (such as the strong correlation between flowering time and biomass in the drought treatment) will also affect the total selection on and potential response to selection of ecophysiological traits. Similarly, it is important to note that the presence of G x E in our experiment indicates that patterns of trait variation and genetic correlation may change under other types of drought, which would also likely affect patterns of selection on drought response.

Complementary genetic studies suggest the genetic correlation between flowering time and WUE may be largely due to pleiotropy affecting both physiology and phenology in *A. thaliana*. Multiple quantitative trait loci (QTL) colocalize for WUE and flowering time (Hausmann et al. 2005; Juenger et al. 2005a). Furthermore, the genes FRIGIDA (McKay et al. 2003; Lovell et al. 2013) and Flowering Locus C (McKay et al. 2003) pleiotropically affect WUE and flowering time in *A. thaliana*. Flowering Locus T, a known flowering time gene, also regulates stomatal opening, a trait that directly affects WUE (Kinoshita et al. 2011). It is unknown if other loci annotated as flowering time genes also have effects on WUE.

Although pleiotropy likely underlies much of the genetic correlation observed in our experiment, it is also possible that local adaptation to climate (Fournier-Level et al. 2011; Hancock et al. 2011) has created linkage disequilibrium between genes that independently affect flowering time and WUE, which would also contribute to this genetic correlation. Along these lines, pleiotropic QTL may actually represent variants at multiple tightly linked genes with each affecting WUE and flowering time independently. Also, in addition to the pleiotropic loci underlying WUE and flowering time, there are also multiple separate QTL affecting each of these traits (e.g., Juenger et al. 2005a; Lovell et al. 2013), some of which act epistatically, further demonstrating their complex genetic basis. To date, most studies of natural variation in flowering time have assumed that genetic loci act via a developmental switch leading to rapid transitions from vegetative to reproductive states. However, the presence of genetic correlations and pleiotropy suggests that future studies would benefit from whole plant, integrative approaches that incorporate interactions with autonomous pathways related to carbon fixation. These efforts may ultimately lead to a better understanding of the transition to flowering.

### Season-ending drought favors drought escape in spring flowering *A. thaliana*

We found selection favoring early flowering and lower WUE under both well-watered and terminal drought conditions. WUE was marginally stronger in the drought treatment; however, when accounting for correlations with WUE plasticity in our focal plasticity analysis, selection on WUE was significantly more negative in the drought treatment. Strikingly, both total and direct selection for earlier flowering were significantly stronger in the drought treatment. There was also significant nonlinear selection on flowering time under drought. These results indicate terminal drought conditions favor drought escape rather than a more conservative avoidance strategy in spring flowering accessions of *A. thaliana* and demonstrate the importance of water availability for the evolution of both flowering time and WUE. Furthermore, these results are consistent with field studies in *A. thaliana* that show selection on flowering time loci is season and environment-specific (Weinig et al. 2003; Korves et al. 2007) and recent population genomic studies on climate as a major force shaping local adaptation among populations of *A. thaliana* (Fournier-Level et al. 2011; Hancock et al. 2011; Lasky et al. 2012).

Although selection favored drought escape in the current study, this panel of spring flowering *A. thaliana* accessions has a range of drought response phenotypes from escape to more avoidance-like (i.e., higher WUE and later flowering). *A. thaliana* grows over a large geographic and climatic range (Banta et al. 2012; Lasky et al. 2012) and environments with more severe, sustained, and/or frequent drought may shift the optimum phenotype more toward drought avoidance. This is important to note, as the current experiment specifically mimics a season-ending drought. Patterns of selection may be substantially different under different experimental conditions (e.g., more sustained drought could favor higher WUE and either intermediate or later flowering). Results of the current experiment are also specific to a spring flowering life history; an experiment designed for and including later flowering and/or vernalization-requiring accessions may draw different conclusions regarding selection on drought response. Moreover, previous work in other species demonstrates that selection on ecophysiological traits such as WUE and flowering time is dependent on the genetic background and environment and is likely balanced with selection driven by resources other than water (see citations in introduction for examples).

### Is WUE plasticity to drought adaptive and/or costly?

Plasticity is typically considered adaptive if the direction of plastic change between environments mirrors the difference in selection on phenotypic traits (Schmitt et al. 1999; e.g., Weinig et al. 2006). However, this view does not formally consider potential costs or benefits of plastic development per se, which may act independently of direct selection on phenotypic trait values and/or alter net selection on plasticity. Quantifying variation in phenotypic plasticity and its relationship to fitness in and across environments can directly test for benefits and costs of plasticity (Tienderen 1991; DeWitt et al. 1998; van Kleunen and Fischer 2005, 2007; Auld et al. 2010).

In the current experiment, WUE (*δ* ^13^C) plastic response to drought was in the opposite direction of selection on WUE, which would suggest that WUE plasticity to drought is maladaptive. However, our genetic selection analyses (which included WUE and WUE plasticity) suggest plasticity per se is either neutral or potentially beneficial, depending on the environment. First, we found that total selection (from selection differentials) on WUE plasticity was not significant in either treatment, suggesting WUE plasticity is neutral overall, neither adaptive nor costly. However, it is important to account for possible correlations between plasticity and mean trait values, as well as with other traits, when interpreting selection on plasticity (van Kleunen and Fischer 2007; Auld et al. 2010). Strong correlations between plasticity and mean trait values can bias selection coefficients (Auld et al. 2010) and possibly result in over- or under-estimating selection on plasticity. In the current analysis, WUE plasticity was correlated with WUE in both treatments, particularly in the drought treatment where higher WUE was strongly correlated with greater plasticity. Because selection favored lower WUE, if this were to introduce a bias, the effect would most likely be to reduce the estimated selection on plasticity. Additionally, because plasticity was (weakly) positively correlated with flowering time in the drought treatment (later flowering correlated with greater plasticity), selection on earlier flowering could cause a similar downward bias in selection on plasticity. Therefore, based on these correlations, we believe our estimates of direct selection on WUE plasticity may be conservative. Moreover, after accounting for these relationships in our multivariate selection analyses, we found that direct selection significantly favored greater WUE plasticity in the drought treatment, but remained neutral in the long season treatment. Our global versus local selection analyses (van Kleunen and Fischer 2005) mirrored these results; greater WUE plasticity was locally favored under drought, but neutral in the long season treatment and globally across treatments. Also, direct selection on WUE plasticity in the drought treatment was in the same direction as plasticity itself, indicating that greater plastic response per se was adaptive after accounting for mean phenotypic trait value. These results indicate that for individuals with the same phenotypic trait value, those that attained their phenotype through greater plastic development were more fit than plants with relatively more fixed development (DeWitt et al. 1998). Given that plants with greater plasticity to drought also had lower WUE under well-watered conditions, it may be that greater plasticity allowed some plants to more fully use water resources when they were plentiful, but then conserve to maximize fitness after the onset of terminal drought.

We found limited evidence for constraints on the evolution of WUE plasticity to drought in *A. thaliana*. First, rank-changing G × E for *δ* ^13^C, moderate between-treatment genetic correlation, and the range of *δ* ^13^C plasticity values indicate there is significant genetic variation in WUE plasticity and potential for future response to selection (Falconer and Mackay 1996; DeWitt et al. 1998; Lynch and Walsh 1998). Second, the most plastic genotypes had the most extreme trait values in both treatments, demonstrating that plastic genotypes are not limited in the range of phenotypes they can produce (DeWitt et al. 1998; van Kleunen and Fischer 2005). Third, in contrast to studies on plasticity for other traits in *A. thaliana,* for example, flowering time plasticity to temperature (Stinchcombe et al. 2004a), incomplete vernalization (Callahan et al. 2005), and apical branch plasticity to density (Weinig et al. 2006), we did not detect fitness costs of WUE plasticity. Finally, the one constraint we observed was the strong genetic correlation between WUE and WUE plasticity, which may limit the evolution of different trait-value/plasticity combinations (Falconer and Mackay 1996; Schluter 1996; Lynch and Walsh 1998). The extent to which this correlation may constrain the independent evolution of WUE plasticity will depend on the extent that mean WUE and WUE plasticity share a genetic basis. Molecular studies have revealed some of the pathways controlling stomatal opening and closing to regulate gas exchange in the Columbia accession of *A. thaliana* (Schroeder et al. 2001; Nilson and Assmann 2007; Acharya and Assmann 2009; Ward et al. 2009). Molecular studies have also identified genes affecting leaf development, with effects on photosynthetic capacity (e.g., Masle et al. 2005) and/or transpiration (e.g., Masle et al. 2005; Boccalandro et al. 2009). Moreover, variation in photosynthetic capacity can be caused by a multitude of possible components and both this and stomatal regulation affect WUE. Future studies will need to determine what subset of these components are responsible for the striking differences in acclimation and adaptation we see within and among crops and wild species.

Overall, our results suggest plasticity may be important for the evolution of drought response in spring flowering *A. thaliana*. Whether plasticity per se is under selection in *A. thaliana* likely depends on the nature of environmental variation experienced by individual genotypes. Global selection on plasticity and the response to selection will be influenced by the frequency and distribution of different environments in nature (note that our global analysis assumes equal frequency of the two environments). *A. thaliana* has a predominantly selfing mating system and multiple individuals within the same population often have the same multilocus haplotype (Bakker et al. 2006). Additionally, different populations within North America often share multilocus haplotypes (Bakker et al. 2006). Therefore, individual genotypes likely experience multiple environments over time and/or across space, and it is possible that selection on plasticity in *A. thaliana* can act both directly on plasticity per se and indirectly through selection on mean trait values (Via et al. 1995). It would be of great theoretical and applied interest to understand which molecular variants underlie this variation in adaptive plasticity.

## Supporting Information

**Table S1**. *Arabidopsis thaliana* accessions used in this experiment. **Table S2.** Standardized nonlinear and correlational selection gradients within the long season and terminal drought treatments.
