# Supplementary material for: Direct and indirect selection on flowering time, water-use efficiency (WUE, δ 13C), and WUE plasticity to drought in Arabidopsis thaliana
Source: Ecol Evol. 2014 Nov 19;4(23):4505–21. doi: 10.1002/ece3.1270 (PMC4264900; doi:10.1002/ece3.1270)
Supplement: Supplementary file 1 — Table S1. Arabidopsis thaliana accessions used in this experiment. Table S2. Standardized nonlinear and correlational selection gradients within the long season and terminal drought treatments. [file ece30004-4505-sd1.docx]

**Appendix Supplementary tables S1 and S2**

**Table S1** *Arabidopsis thaliana* accessions used in this experiment

**Table S2** Standardized nonlinear and correlational selection gradients within the long season and terminal drought treatments

Standardized nonlinear (γ_ii_) and correlational (γ_ij_) selection gradients were estimated in a full model containing all linear effects. Nonlinear gradients (γ_ii_) and associated standard errors and confidence intervals were doubled. *N* = 206 for both treatments. Significant values are in bold**.**
